# Adrenergic receptors and metabolism: role in development of cardiovascular disease

**DOI:** 10.3389/fphys.2013.00265

**Published:** 2013-10-03

**Authors:** Michele Ciccarelli, Gaetano Santulli, Valeria Pascale, Bruno Trimarco, Guido Iaccarino

**Affiliations:** ^1^Department of Medicine and Surgery, University of SalernoSalerno, Italy; ^2^Center for Translational Medicine, Department of Pharmacology, Temple University of PhiladelphiaPA, USA; ^3^Department of Physiology and Cellular Biophysics, Columbia University in the City of New YorkNew York, NY, USA; ^4^Department of Advanced Biomedical Sciences“Federico II” University of NaplesItaly; ^5^IRCCS, Multimedica Research HospitalMilan, Italy

**Keywords:** cardiac metabolism, beta adrenergic system, heart failure, GRKs, mitochondria

## Abstract

Activation of the adrenergic system has a profound effects on metabolism. Increased circulating catecholamine and activation of the different adrenergic receptors deployed in the various organs produce important metabolic responses which include: (1) increased lipolysis and elevated levels of fatty acids in plasma, (2) increased gluconeogenesis by the liver to provide substrate for the brain, and (3) moderate inhibition of insulin release by the pancreas to conserve glucose and to shift fuel metabolism of muscle in the direction of fatty acid oxidation. These physiological responses, typical of the stress conditions, are demonstrated to be detrimental for the functioning of different organs like the cardiac muscle when they become chronic. Indeed, a common feature of many pathological conditions involving over-activation of the adrenergic system is the development of metabolic alterations which can include insulin resistance, altered glucose and lipid metabolism and mitochondrial dysfunction. These patterns are involved with a variably extent among the different pathologies, however, they are in general strictly correlated to the level of activation of the adrenergic system. Here we will review the effects of the different adrenergic receptors subtypes on the metabolic variation observed in important disease like Heart Failure.

## Introduction

Heart function relies to a great extent on cardiac muscle oxidative metabolism. Given the high mitochondrial content of this tissue, cardiac muscle generates ATP almost exclusively (about 90%) through oxidative phosphorylation (Stanley et al., 2005) by using different metabolic substrates. Indeed, cardiac muscle possesses a metabolic flexibility or plasticity, allowing it to maintain its function during stressful conditions. In the adult heart the major pathway for ATP production is fatty acid oxidation while the relative contribution of glucose increases during stress or injury, such as exercise or ischemia (Bing et al., 1954; Wisneski et al., 1987). Thus, it is not surprising that an impairment of cardiac muscle energy metabolism represents an important risk factor for the development of cardiac diseases (Stanley et al., 2005; Neubauer, 2007). Indeed, under pathological conditions, the heart exhibits a severe malfunction of different metabolic pathways, such as the tricarboxylic acid (TCA) cycle and b-oxidation (Stanley et al., 2005; Neubauer, 2007). This metabolic remodeling is characterized by a lower oxidative capacity, contractile dysfunction and cardiac muscle insulin resistance (Stanley et al., 2005; Neubauer, 2007). Different therapeutic strategies have been undertaken to modulate metabolic pathways in the failing heart, though it remains controversial whether targeting glucose vs. fatty acid metabolism individually or combined represents a better approach to improve metabolic flexibility and cardiac function (Kolwicz and Tian, 2009; Ardehali et al., 2012). Surely, the importance of a preserved metabolism includes not only an efficient energy supply needed for myocardium to accomplish his contractile function but also protection against oxidative stress (Neubauer, 2007), which is involved in the remodeling and progression of the disease.

## Cardiac metabolic dysfunction during HF

Animal models of HF have evidenced excessive uptake and myocardial free fatty acid (FFA) accumulation with reduced glucose utilization (Lommi et al., 1998; Taylor et al., 2001) and in both animal models of HF and in human disease these metabolic alterations reduce myocardial oxygen efficiency and lead to a depletion of intracellular ATP (Neubauer et al., 1992; Taylor et al., 2001). The importance of a preserved glucose cardiac utilization may be related to its higher efficiency in terms of ATP production and molecule of oxygen consumed, with consequently reduced oxygen wastage and reactive oxygen species (ROS) production, as compared to FFA. Insulin receptor signaling is critically involved in increasing glucose uptake in the myocardium and cardiac insulin resistance contributes to the development of left ventricular (LV) dysfunction by reducing cardiac efficiency through metabolic shift toward fatty acids utilization (Peterson et al., 2004b). Indeed, a profound state of insulin resistance has been found in the hearts of *ob/ob* mice and the ability of these hearts to modulate substrate utilization in response to insulin and changes is altered (Mazumder et al., 2004). Accordingly, normalization of cardiac metabolism by overexpressing a human GLUT4 transgene in mice with cardiac insulin resistance recovered the altered cardiac function observed in these animals (Belke et al., 2000; Semeniuk et al., 2002). Therefore, these studies indicate that cardiac insulin resistance reduces the metabolic efficiency of the heart, which leads to a contractile dysfunction. Moreover, insulin resistance is a known and recognized phenomenon leading to HF (Boudina et al., 2009) as seen in positron emission tomographic (PET) studies showing that the failing human myocardium has reduced glucose uptake in favor of FFA uptake (Witteles et al., 2004). Several hypotheses have been proposed to explain the association between altered cardiac metabolism, insulin resistance, and HF, and among these there is a strong correlation with neurohormonal activation (Kostis and Sanders, 2005; Zucker, 2006), which increases plasma FFA levels, inhibits insulin receptor signaling and causes the loss of myocyte glucose uptake (Opie and Sack, 2002). As known neurohormonal activation includes over-activation of the adrenergic and RAAS system, and we will here specifically focus on the adrenergic mechanisms that underlie altered myocardial metabolism and insulin resistance in HF.

### Sympathetic nervous system and cardiac metabolism: role of the β adrenergic receptors and related signaling

The Sympathetic Nervous System (SNS) is maladaptively activated in response to a chronic reduction in cardiac output and it is characterized by an increased secretion and reduced cardiac catecholamine reuptake (Eisenhofer et al., 1996). The effects of the catecholamine incretion on the cardiac metabolism are mediated by both central and peripheral mechanisms. For example, increased catecholamines have directly detrimental effects on the heart, which cause marked enzyme loss as an index of diffuse myocardial damage, and substantial oxygen-wastage even in the absence of FFA in the perfusate (Opie et al., 1979). Furthermore, norepinephrine promotes both coronary vasoconstriction and increased plasma FFA levels (Paolisso et al., 1991), which further promote oxygen-wastage (Sasaoka et al., 2006). Infusion studies in volunteers support a role for increased norepinephrine levels in HF as a cause of elevated plasma FFA (Sasaoka et al., 2006). In turn, FFAs reciprocally augment SNS activity, at least in normal controls. In human skeletal muscle, a dose-response relationship exists between plasma FFA (Santomauro et al., 1999; Peterson et al., 2004a; Banerjee and Peterson, 2007) and defects in insulin signaling (Belfort et al., 2005). This may in part be caused by FFA-mediated activation of protein kinase C, which phosphorylates insulin receptors and results in reduced capillary opening and reduced myocyte glucose import (Itani et al., 2002; Wagenmakers et al., 2006). Moreover, also locally activated SNS appear to be relevant in the altered cardiac metabolism. With use of PET with a norepinephrine analog and 18F-fluorodeoxyglucose, myocardial segments with LV dysfunction have reduced presynaptic norepinephrine reuptake and myocardial glucose uptake in relation to less impaired myocardial segments in the same patients (Mongillo et al., 2007). Thus, after control for confounding variables, altered metabolism and IR directly relate to local SNS activity. As known, the adverse effects of the SNS on the heart are mediated by the adrenergic receptors (AR), however, extensive research has indicated that the various subtypes, in particular β_1_ and β_2_ ARs, are differently involved in pathophysiology of HF and so it is likely to be the same for modifications of cardiac metabolism observed during disease. Indeed, β_1_- and β_2_-AR regulate different signal pathways, resulting in different outcomes on cardiac function. Stimulation of β_1_- and β_2_-adrenoceptors can induce the activation of the stimulatory G protein (G_αs_)/adenylylcyclase (AC)/cAMP/cAMP-dependent protein kinase A (PKA) signaling pathway, which consequently leads to the phosphorylation of several target proteins within the cardiac myocyte, such as phospholamban, L-type calcium channel and troponin I (Woo and Xiao, 2012). Nonetheless, this signal pathway is the main mechanism by which β_1_- rather than β_2_ adrenoceptors regulates cardiac contractility/relaxation and rate (Baruscotti et al., 2010; Woo and Xiao, 2012). In contrast, the β_2_ AR has been shown to regulate an alternative signaling pathway through activation of the inhibitory G protein (G_αi_) and the heterodimer formed by the β and γ subunits of the G protein (G_βγ_) (Zhu et al., 2011). Besides the inhibition of AC, the main signal pathway regulated by β_2_-AR through G_αi_/G_βγ_ appears to be the phosphatidylinositol-3kinase (PI3K) signaling cascade, although other proteins such as the AMP-dependent protein kinase (AMPK), mammalian target of rapamycin (mTOR) and extracellular signal-regulated kinase1and 2 (ERK1/2) have recently been proposed as novel targets of β_2_-AR (Zhang et al., 2011). About the effects of adrenergic system on metabolism, it is known that sustained beta adrenergic stimulation induce insulin resistance (Cipolletta et al., 2009) and in this context the β_2_ adrenergic receptor appears to have a main role in overall glucose homeostasis by acting on pancreatic islet hormone secretion, liver and muscle glucose transport metabolism (Usui et al., 2005; Shahid and Hussain, 2007; Garcia-Guerra et al., 2010). At cardiac level several studies have raised the possibility of using selective β_2_-agonists as potential modulators of cardiac muscle energy metabolism. Short- and long-term stimulation of the β_2_-AR has been associated with the modulation of fatty acid and glucose metabolism (Philipson, 2002). Indeed, acute treatment of myocytes *in vitro* or skeletal muscle *ex vivo* with β_2_ agonists induces a significant increase in glucose uptake, reaching comparable levels to insulin stimulation (Nevzorova et al., 2002, 2006; Ngala et al., 2008). A putative mechanism for these evidence would involve the activation of PI3K and its downstream signal pathway (Zhu et al., 2001; Jo et al., 2002; Perez-Schindler et al., 2011; Zhang et al., 2011) and in particular the phosphorylation and inactivation of TBC1D4 (also known as Akt substrate of 160 kDa, AS160) by Akt (Sakamoto and Holman, 2008). TBC1D4 inhibit the translocation of the glucose transporter type4 (GLUT4) from intracellular vesicles to the plasmamembrane, hence an increase in TBC1D4 phosphorylation would consequently enhance glucose uptake (Sakamoto and Holman, 2008). Moreover, TBC1D4 is also targeted by AMPK, which represent a key mechanism in the regulation of insulin-independent glucose uptake (Sakamoto and Holman, 2008; Maarbjerg et al., 2011). Consistent with the potential role of β_2_-AR in glucose metabolism, high levels of AMPK phosphorylation and activity in response to β_2_-AR stimulation (Li et al., 2010; Perez-Schindler et al., 2011) have been found in cardiomyocytes, potentially as consequence of an increase in the AMP/ATP ratio, or activation of upstream AMPK kinases (Hardie et al., 2012). Different results, however, have been found in presence of an enhanced β_2_-AR signaling by means of stable overexpression in non-cardiac cells, where, *in vitro* studies shows impaired glucose extraction later on insulin stimulation (Cipolletta et al., 2009). Moreover “*in vivo*” studies demonstrated a greater efficiency of carvedilol, a non-selective β-AR antagonist, in ameliorating myocardial insulin sensitivity and glucose extraction in animal model of HF as compared to metoprolol, a selective β_1_-AR antagonist, indicating that antagonism of β_2_AR positively regulates cardiac glucose metabolism in an animal model HF (Nikolaidis et al., 2006). These conflicting results may rely on the different models used for studies and/or to differences in acute and chronic stimulation of the β_2_AR. While acute activation of the receptor can favor glucose uptake by increasing GLUT4 translocation to the plasmamembrane, chronic adrenergic stimulation, as seen during HF, would be rather detrimental by up or down regulation of specific intracellular molecules. Recent discoveries have found in the kinase of the G protein coupled receptor type 2 (GRK2) a potential molecular link between chronic adrenergic stimulation and development of altered myocardial metabolism observed during HF (Ciccarelli et al., 2011). So far, the implications of GRK2 upregulation during HF have been exclusively considered for the detrimental effects on β AR signaling and cardiac inotropism (Koch et al., 1995), however, studies in non-cardiac cells have shown ability to interact with different substrates belonging to different cellular pathways such as insulin signaling, and, of note, to mediate insulin resistance produced by enhanced β_2_-AR receptor signaling (Cipolletta et al., 2009). In the failing heart GRK2 is upregulated (Iaccarino et al., 2005) and affects myocardial glucose uptake at the early stages of the disease, before cardiac dilation and reduced function are evident, indicating that metabolic modifications are relevant in the progression of HF and to be the molecular link between over-activation of the adrenergic system and the altered glucose uptake during HF (Ciccarelli et al., 2011). Recent studies have also indicated that GRK2 is also able to localize into mitochondria, but this role in the global cellular physiology is not completely clear. Indeed, different cellular models have shown different behaviors of GRK2 when it localizes into mitochondria. Specifically, Fusco et al. have indicated that GRK2 is protective for the cell by increasing ATP production and promoting mitogenesis (Fusco et al., 2012). As known, GRK2 shuttles from cytosol to plasmamembrane, by anchoring to free Gβγ through its pleckstrin homology and binding domains within the carboxyl terminus, following both catecholamine and insulin stimulation. Similarly, ischemia causes acute cellular and mitochondrial accumulation of GRK2 (Fusco et al., 2012), an effect reverted by oxygen restoration, but, surprisingly it preserves ATP loss and induces mitochondria biogenesis after ischemia/reperfusion, indicating a protective effect of GRK2 for mitochondria after acute stress.

These data are supported by the importance of GRK2 for embryonic cardiac development (Jaber et al., 1996) and, in adult life, by the early eccentric dilation of the heart and the vascular inflammation induced by selective GRK2 removal in the myocardium and endothelium, respectively (Brunn et al., 2006; Ciccarelli et al., 2013). On the contrary, Chen et al. have also shown that elevated level of GRK2 activates a pro-death signal through its localization into mitochondria during stress conditions (Chen et al., 2013).

**Figure 1:**
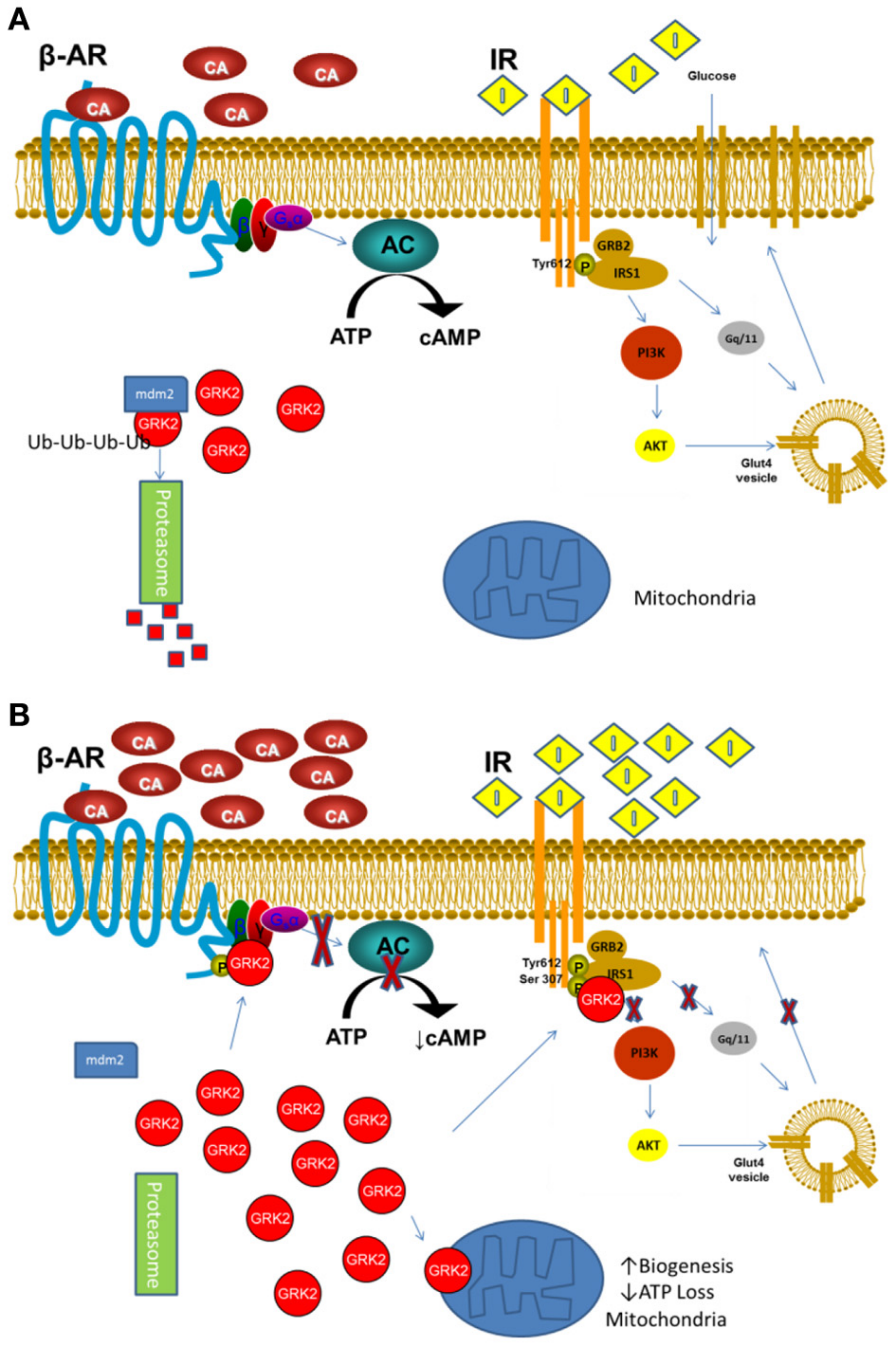
**(A)** In basal or low stimulation conditions GRK2 is mostly cytosolic and its level are tightly regulated by mdm2 ubiquitination and degradation through the Proteasome. **(B)** Following acute or excessive stimulation by catecholamines or Insulin or hypoxia, GRK2 detaches from mdm2 and quickly accumulates in the cell, moving to membranes where desensitizes β-AR and IR. Moreover, it can translocates to the mitochondria, reducing ATP loss and inducing mitochondria biogenesis (β-AR, β Adrenergic Receptor; IR, Insulin Receptor; CA, Catecholamine; I, Insulin).

This discrepancy could be related to the model and timing used in different studies, however, in a synthetic view of the latest GRK2 studies, we could advance the idea that the inhibitory property of GRK2 could be useful in acute phase of stress/stimulation, when the cytosolic molecule shuttles to the plasmamembrane and mitochondria, setting cellular activity in a resting phase and less susceptible to the stress and/or excess of signaling. However, in a perpetuating situation, the increased activity/level of GRK2 and chronic reduction of β adrenergic and insulin signaling becomes detrimental for cell function and survival (Figures 1A,B). Further studies will be needed to evaluate this hypothesis; nevertheless, several reports already indicate GRK2 as a potential target for treatment of both altered metabolism and contractility during HF.

## Conclusions and perspectives

Modification of cardiac metabolism is a fascinating field of investigation for development of new strategies for treatment of myocardial dysfunction. This consideration is based on the evidence that an efficient metabolic supply is needed for the single cardiomyocyte to accomplish its contractile function and efficient utilization is also important to counteract oxygen wastage and increased ROS on which relies progression of the disease. Current available compounds acting on the β ARs seem to be effective for these goals, however, identification of specific target, as GRK2, clearly involved in regulation of cardiac contractility, remodeling and metabolism would prepare the ground for a more efficient therapy aimed to ameliorate patients prognosis and outcome.

### Conflict of interest statement

The authors declare that the research was conducted in the absence of any commercial or financial relationships that could be construed as a potential conflict of interest.
